# Supplementary material for: Reversible surface modifications of functional proteins for accelerated cytosolic delivery via cell-penetrating peptide clusters
Source: Nat Commun. 2026 Mar 2;17:3341. doi: 10.1038/s41467-026-70054-6 (PMC13065835; doi:10.1038/s41467-026-70054-6)
Supplement: Supplementary file 1 — Supplementary Information [file 41467_2026_70054_MOESM1_ESM.pdf]

# Supplementary Information

# Reversible surface modifications of functional proteins for accelerated cytosolic delivery via cell-penetrating peptide clusters

Xiao Hua[1,2,3#], Yanyan Guo[3#], Pincheng Li[1#], Yu Wang[1#], Xiaona Han[1], Junyou Chen[1], Junjiang Li[1], Guochao Chu[1,2], Jing Shi[3], Lei Liu[2*], Yi-Ming Li[1,4*]

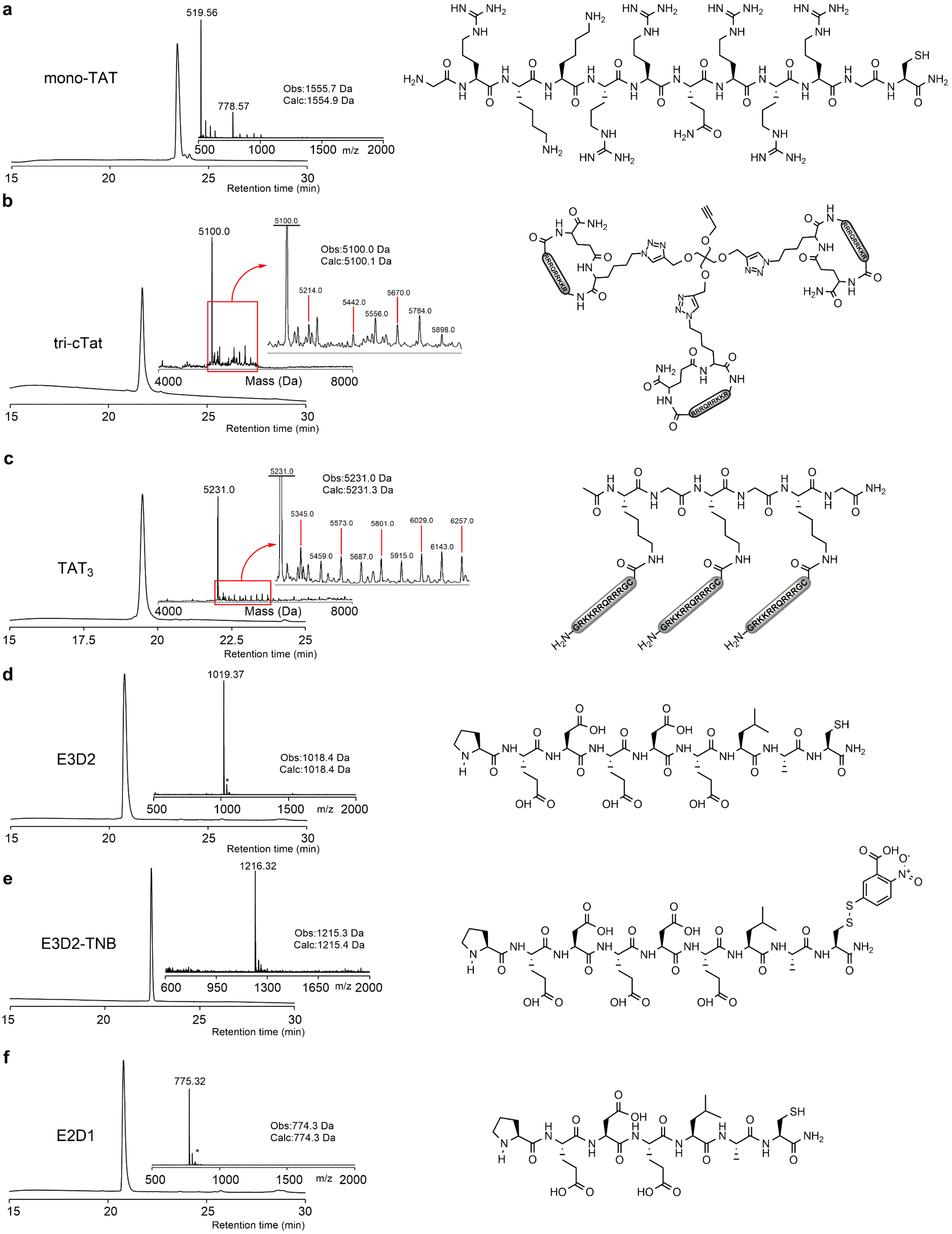
a
mono-TAT
519.56
778.57
Obs:1555.7 Da
Calc:1554.9 Da
500 1000 1500 m/z 2000
15 20 25 30
Retention time (min)
b
tri-cTat
5100.0
Obs:5100.0 Da
Calc:5100.1 Da
5214.0
5442.0
5556.0
5670.0
5784.0
5898.0
4000 Mass (Da) 8000
15 20 25 30
Retention time (min)
c
TAT3
5231.0
Obs:5231.0 Da
Calc:5231.3 Da
5345.0
5459.0
5573.0
5687.0
5801.0
5915.0
6029.0
6143.0
6257.0
4000 Mass (Da) 8000
15 17.5 20 22.5 25
Retention time (min)
GRKKRRQRRRGC
d
E3D2
1019.37
Obs:1018.4 Da
Calc:1018.4 Da
500 1000 1500 m/z 2000
15 20 25 30
Retention time (min)
e
E3D2-TNB
1216.32
Obs:1215.3 Da
Calc:1215.4 Da
600 950 1300 1650 m/z 2000
15 20 25 30
Retention time (min)
f
E2D1
775.32
Obs:774.3 Da
Calc:774.3 Da
500 1000 1500 m/z 2000
15 20 25 30
Retention time (min)

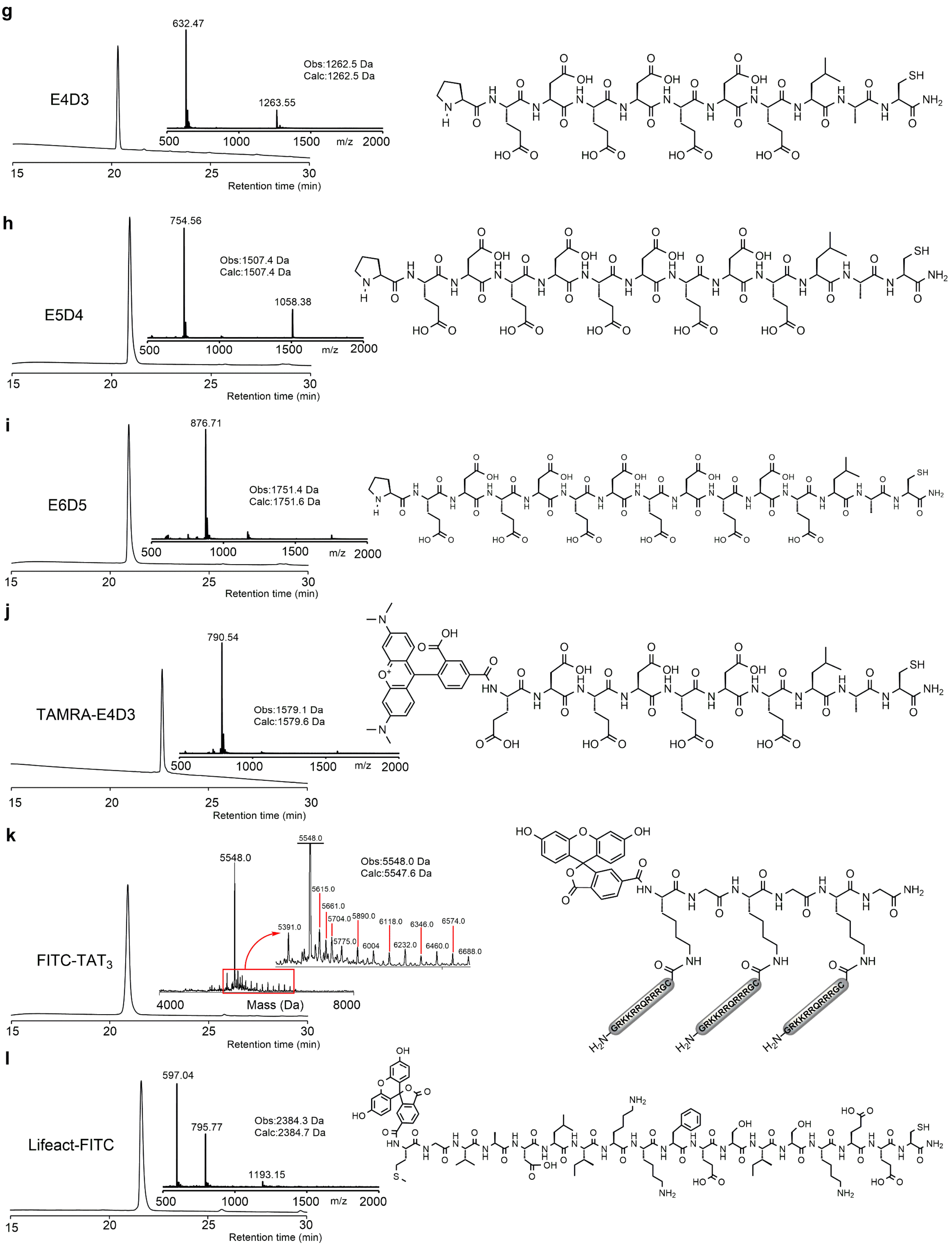
g
E4D3
632.47
1263.55
Obs:1262.5 Da
Calc:1262.5 Da
500 1000 1500 m/z 2000
15 20 25 30
Retention time (min)
h
E5D4
754.56
1058.38
Obs:1507.4 Da
Calc:1507.4 Da
500 1000 1500 m/z 2000
15 20 25 30
Retention time (min)
i
E6D5
876.71
Obs:1751.4 Da
Calc:1751.6 Da
500 1000 1500 m/z 2000
15 20 25 30
Retention time (min)
j
TAMRA-E4D3
790.54
Obs:1579.1 Da
Calc:1579.6 Da
500 1000 1500 m/z 2000
15 20 25 30
Retention time (min)
k
FITC-TAT3
5548.0
Obs:5548.0 Da
Calc:5547.6 Da
5391.0 5615.0 5661.0 5704.0 5775.0 5890.0 6004 6118.0 6232.0 6346.0 6460.0 6574.0 6688.0
4000 Mass (Da) 8000
15 20 25 30
Retention time (min)
GRKKRRQRRRGC
l
Lifeact-FITC
597.04
795.77
1193.15
Obs:2384.3 Da
Calc:2384.7 Da
500 1000 1500 m/z 2000
15 20 25 30
Retention time (min)

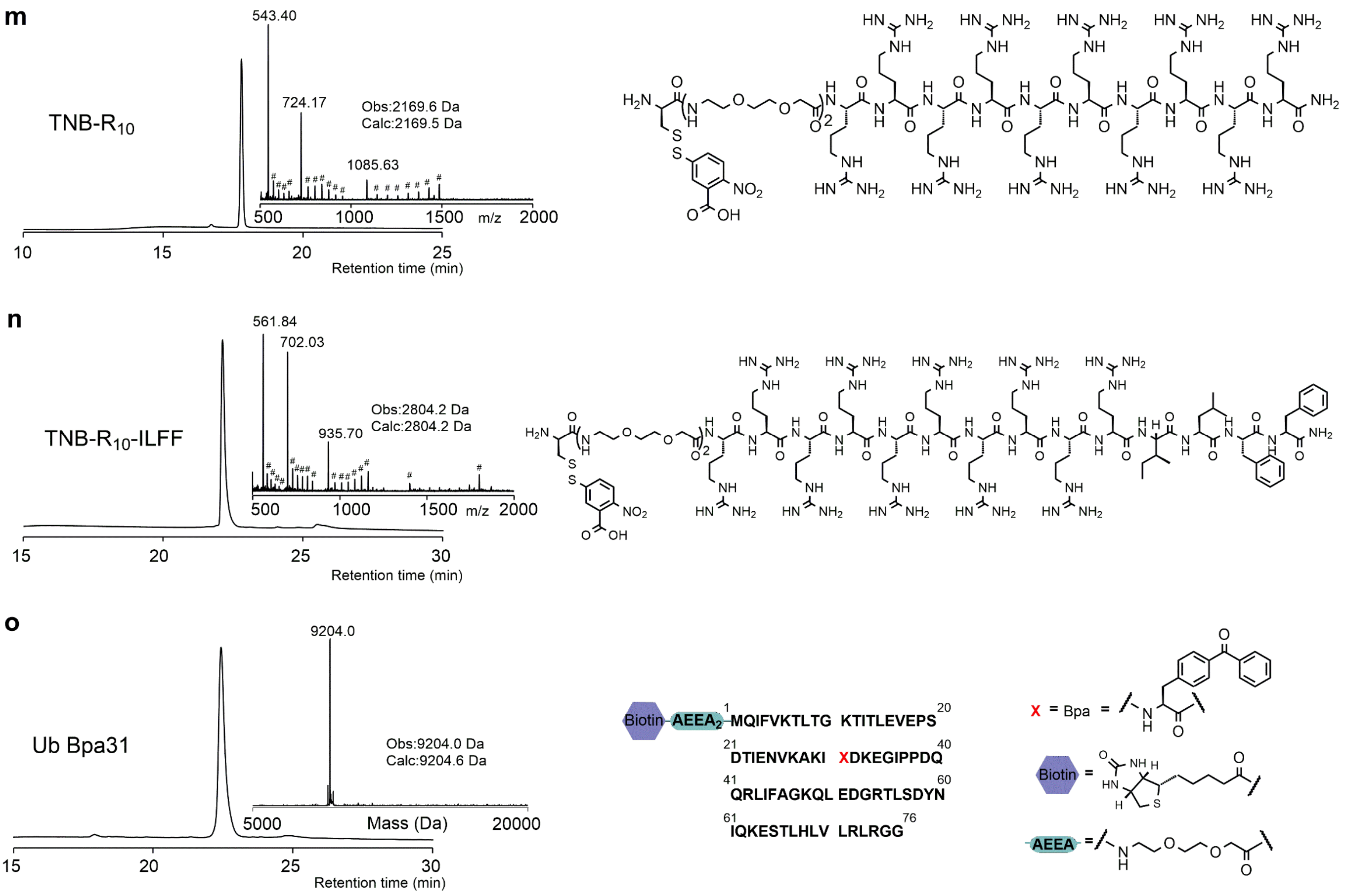


**Supplementary Fig. 1. RP-HPLC and ESI-MS of peptides used in this study. a,** mono-TAT. **b,** tri-cTat. **c,** TAT$_3$. **d,** E3D2. **e,** E3D2-TNB. **f,** E2D1. **g,** E4D3. **h,** E5D4. **i,** E6D5. **j,** TAMRA-E4D3. **k,** FITC-TAT$_3$. **l,** Lifeact-FITC. **m,** TNB-R$_{10}$. **n,** TNB-R$_{10}$-ILFF. **o,** Ub Bpa31. The TFA addition uses either the symbol "#" or a specific molecular weight, which the molecular weight is an integer multiple of 114 Da greater than the expected value. "*" corresponded to sodium adducts.

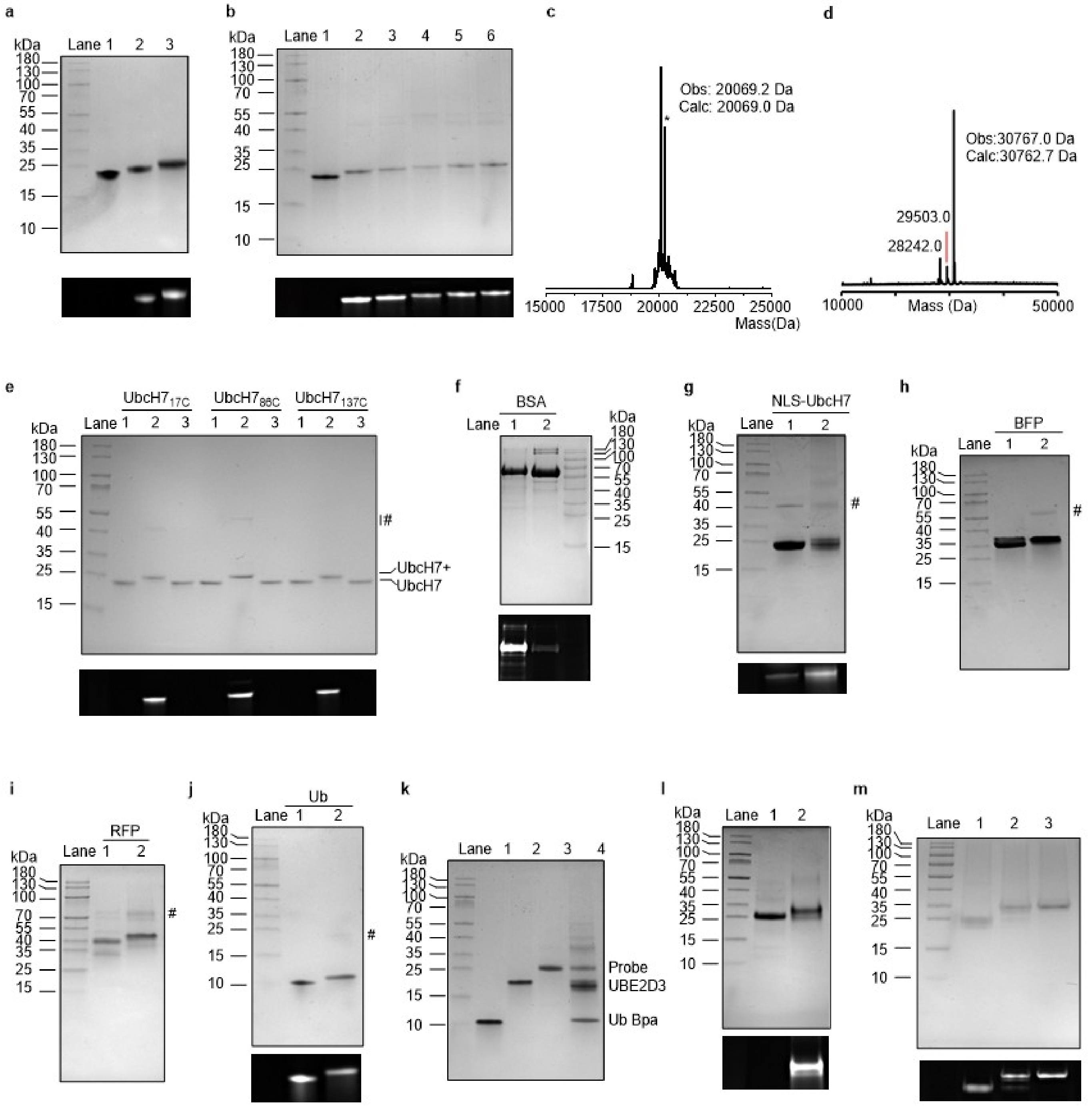


**Supplementary Fig. 2 Characterization of proteins and their adducts. a,** SDS-PAGE gel, stained with Coomassie and fluorescence imaging of UbcH7 samples. Lane 1: UbcH7$_{86C}$; lane 2: UbcH7$_{86C}$-T; lane 3: UbcH7$_{86C}^{E3D2+}$-T. **b,** SDS-PAGE gel, stained with Coomassie and fluorescence imaging of UbcH7$_{86C}$ engineered with different anionic peptides. Lane 1: UbcH7$_{86C}$; lane 2: UbcH7$_{86C}^{E2D1+}$-T; lane 3: UbcH7$_{86C}^{E3D2+}$-T; lane 4: UbcH7$_{86C}^{E4D3+}$-T; lane 5: UbcH7$_{86C}^{E5D4+}$-T; lane 6: UbcH7$_{86C}^{E6D5+}$-T. **c,** Deconvoluted mass characterization of UbcH7$^{E4D3+}$. "*" corresponds to a molecular weight of 20247.2 Da, denoting 178 Da greater than the desired mass attributed to gluconoylation of the N-terminus during protein expression. **d,** Deconvoluted mass characterization of E2-Ub$^{+}$ probe. Molecular weights of 28242 and 29503 correspond to minor products featuring conjugation with one or two E4D3 tags, respectively. **e,** SDS-PAGE gel stained with Coomassie and fluorescence imaging of UbcH7$_{17C}^{+}$-T, UbcH7$_{86C}^{+}$-T and

$UbcH7_{137C}{}^{+}$-T. Lane 1: UbcH7 mutant; lane 2: UbcH7 mutant$^{+}$-T; lane 3: UbcH7 mutant$^{+}$-T incubated with 50 mM DTT. **f,** SDS-PAGE gel, stained with Coomassie and fluorescence imaging of BSA samples. Lane 1: BSA-T; lane 2: BSA$^{+}$-T. **g,** SDS-PAGE gel, stained with Coomassie and fluorescence imaging of NLS-UbcH7 samples. Lane 1: NLS-UbcH7-FITC; lane 2: NLS-UbcH7$^{+}$-FITC. **h,** SDS-PAGE gel stained with Coomassie of BFP samples. Lane 1: BFP; lane 2: BFP$^{+}$. **i,** SDS-PAGE gel stained with Coomassie of RFP samples. Lane 1: RFP; lane 2: RFP$^{+}$. **j,** SDS-PAGE gel, stained with Coomassie and fluorescence imaging of Ub samples. Lane 1: Ub-T; lane 2: Ub$^{+}$-T. **k,** SDS-PAGE gel stained with Coomassie of UBE2D3-Ub Bpa probe samples. Lane 1: Ub Bpa 31; lane 2: UBE2D3 C85K S22R; lane 3: Purified UBE2D3-Ub Bpa probe; lane 4: the reaction mixture. **l,** SDS-PAGE gel, stained with Coomassie and fluorescence imaging of UBE2D3-Ub Bpa probe samples. Lane 1: UBE2D3-Ub Bpa probe; lane 2: UBE2D3-Ub Bpa probe$^{+}$-T. **m,** SDS-PAGE gel, stained with Coomassie and fluorescence imaging of $TAT_3$-$UbcH7_{86C}$-T samples. Lane 1: $UbcH7_{86C}$-T; lane 2: the reaction mixture of $UbcH7_{86C}$-T and $TAT_3$-TNB; lane 3: Purified $TAT_3$-$UbcH7_{86C}$-T. “#” denotes the dimer of proteins. Source data are provided as a Source Data file.

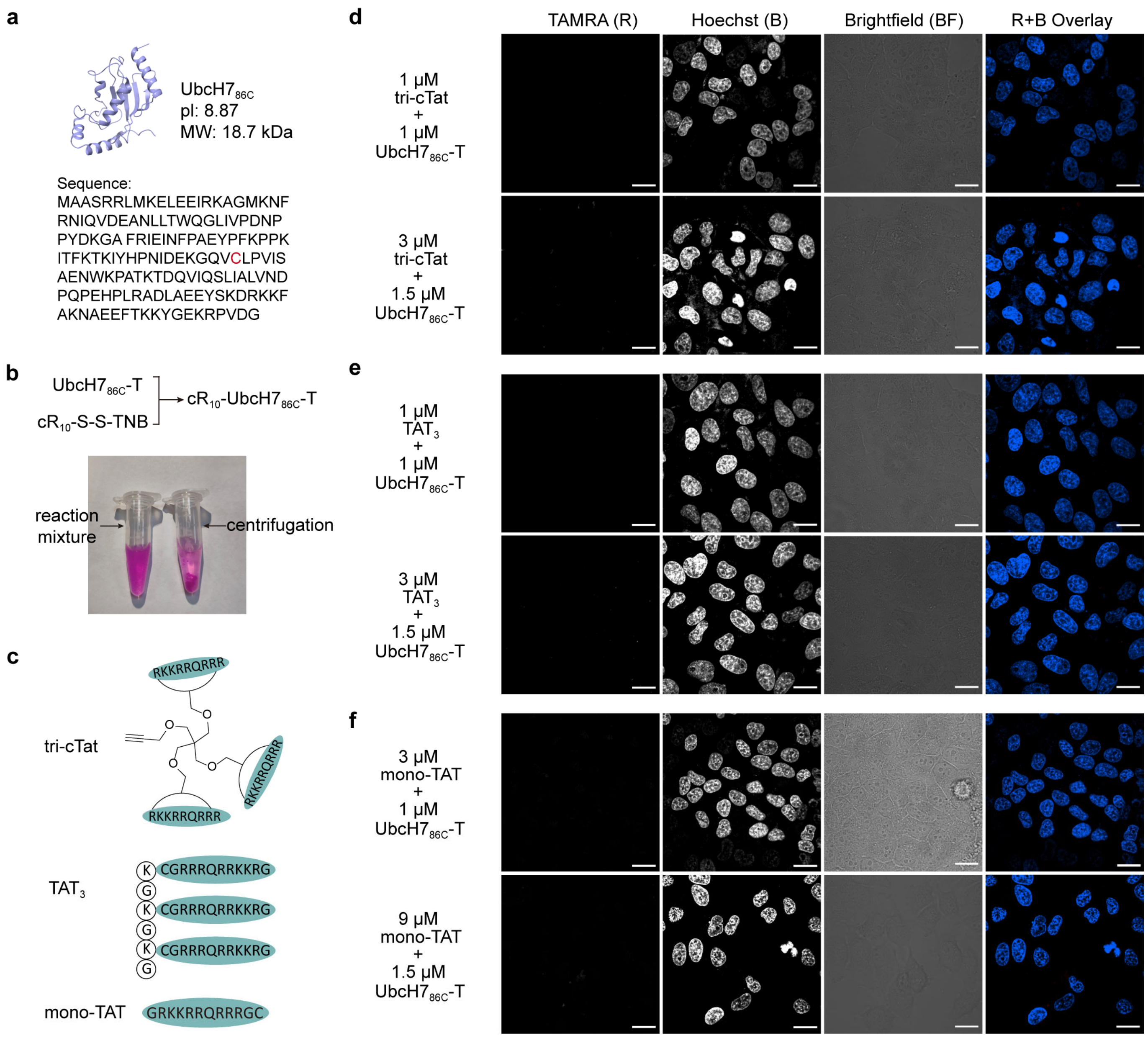


**Supplementary Fig. 3 Cellular uptake of Ubch7$_{86C}$-T using TATs. a,** Structure and sequence of UbcH7$_{86C}$. **b,** Experimental observations of the reaction between UbcH7$_{86C}$-T and cR$_{10}$-S-S-TNB. **c,** Structure and sequence of three types of TATs. **d,** Representative images of HepG2 cells treated with UbcH7$_{86C}$-T in the presence of tri-cTat for 30 minutes at 37 °C. **e,** Representative images of HepG2 cells treated with UbcH7$_{86C}$-T in the presence of TAT$_3$ for 30 minutes at 37 °C. **f,** Representative images of HepG2 cells treated with UbcH7$_{86C}$-T in the presence of mono-TAT for 30 minutes at 37 °C. The images shown in Supplementary Fig. 3d-3f are representative of independent biological replicates (n = 3). "B", "R", and "BF" stand for Hoechst (blue), TAMRA (red), and brightfield signals, respectively. Scale bars, 20 μm.

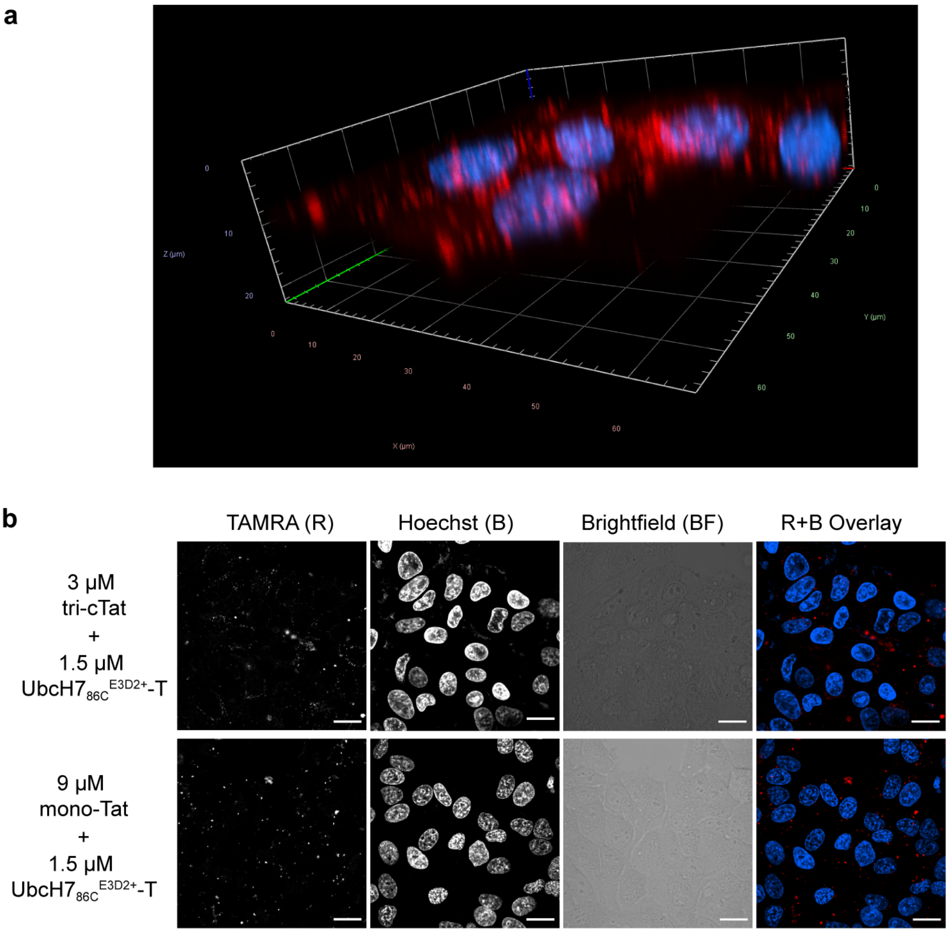


**Supplementary Fig. 4 Cellular uptake of UbcH7$_{86C}^{E3D2+}$-T using tri-cTat and mono-Tat. a,** Z-stack image of HepG2 cells. HepG2 cells were treated with 1.5 μM UbcH7$_{86C}^{E3D2+}$-T and 3 μM $TAT_3$ for 30 minutes at 37 °C. **b,** Representative images of HepG2 cells incubated with 1.5 μM UbcH7$_{86C}^{E3D2+}$-T in the presence of 3 μM tri-cTat or 9 μM mono-TAT for 30 minutes at 37 °C. The images shown are representative of independent biological replicates (n = 3). "B", "R", and "BF" stand for Hoechst (blue), TAMRA (red), and brightfield signals, respectively. Scale bars, 20 μm.

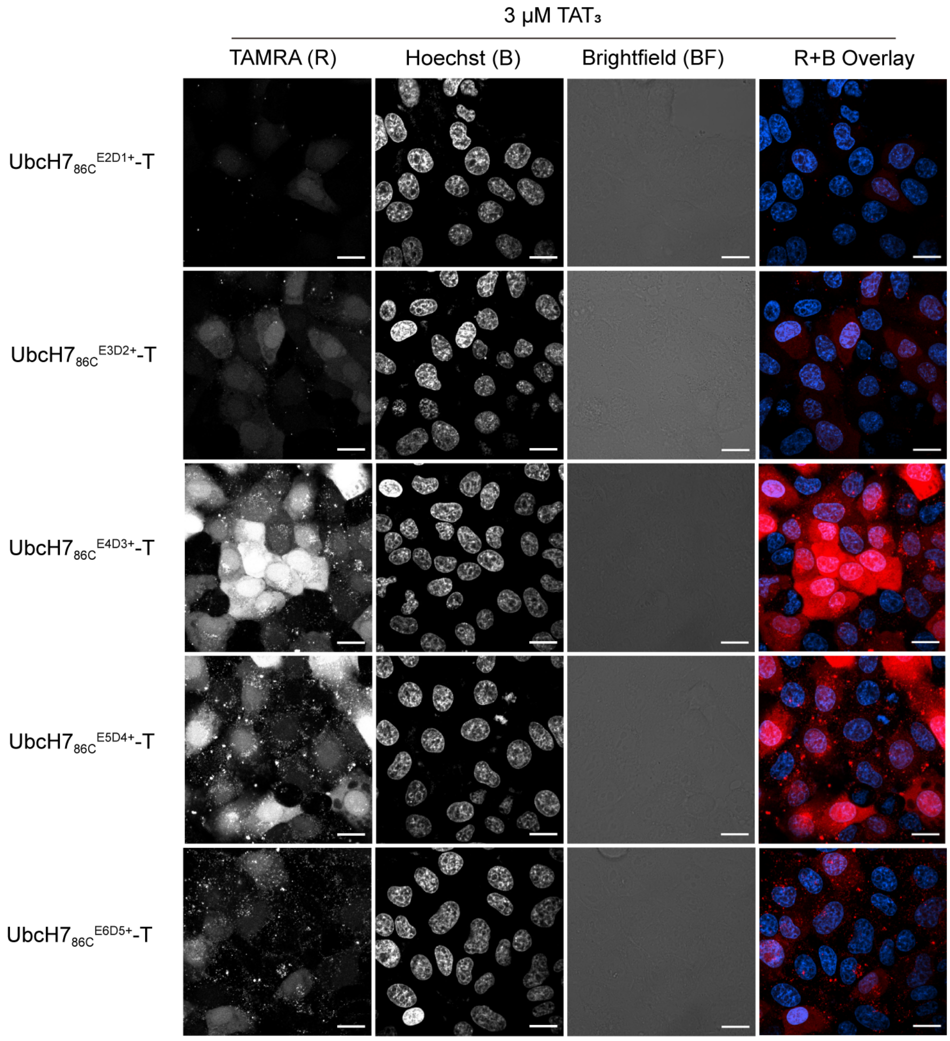


**Supplementary Fig. 5 Full set of confocal microscopy images of Fig. 3a.** HepG2 cells were treated with 1.5 µM five kinds of anionic peptide-engineered UbcH7$_{86C}$-T, respectively, in the presence of 3 µM TAT$_3$ for 30 min at 37 °C, followed by washing with PBS containing heparin for imaging. The images shown are representative of independent biological replicates (n = 3). "B", "R", and "BF" stand for Hoechst (blue), TAMRA (red), and brightfield signals, respectively. Scale bars, 20 µm.

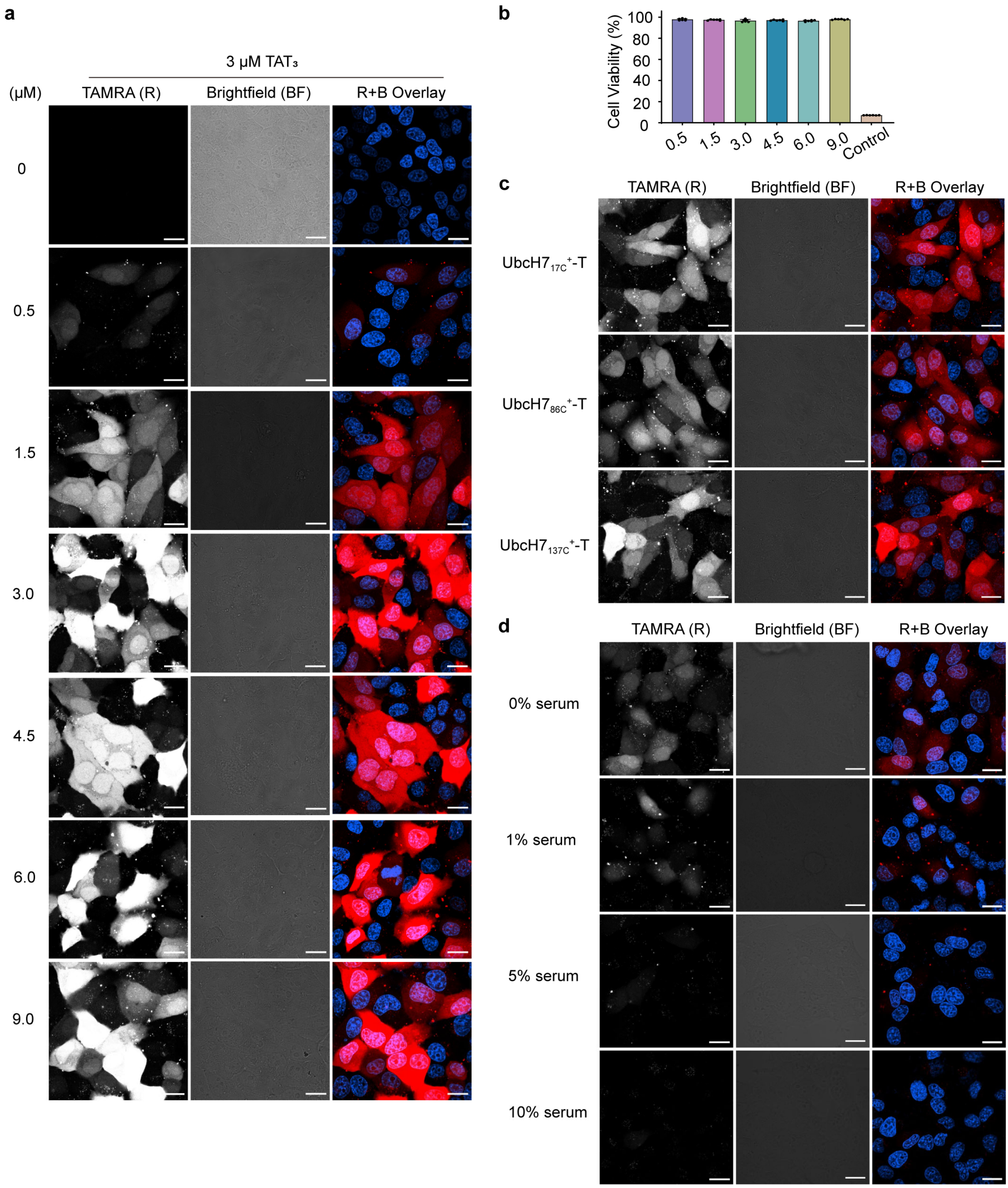


**Supplementary Fig. 6 Titration of UbcH7$_{86C}^{+}$-T into cells with constant concentration of TAT$_3$. a,** Confocal microscopy images of HepG2 cells treated with different concentrations of UbcH7$_{86C}^{+}$-T in the presence of 3 μM TAT$_3$ for 30 minutes at 37 °C, followed by washing with PBS containing heparin for imaging. **b,** Cell viability detected by a CCK-8 assay after treatment of HepG2 cells with different concentrations of UbcH7$_{86C}^{+}$-T in the presence of 3 μM TAT$_3$ for 30 minutes in serum-free DMEM. Treatment with 0.64% phenol was used as a control.

The results are the average of three biological replicates and presented as the mean ± standard deviation. **c,** Representative images of HepG2 cells treated with 3 μM $TAT_3$ and 1.5 μM E4D3 peptide-engineered UbcH7-T, where the modification sites were at the N-terminus, mid-terminus and C-terminus of UbcH7 ($UbcH7_{17C}$, $UbcH7_{86C}$ and $UbcH7_{137C}$), respectively, for 30 minutes at 37 °C, followed by washing with PBS containing heparin for imaging. **d,** Cellular uptake of $UbcH7_{86C}^{+}$-T in the presence of serum. HepG2 cells were incubated with the corresponding samples, followed by washing with PBS containing heparin for imaging. The images shown in Supplementary Fig. 6a, 6c and 6d are representative of independent biological replicates (n = 3). "B", "R", and "BF" stand for Hoechst (blue), TAMRA (red), and brightfield signals, respectively. Scale bars, 20 μm. Source data are provided as a Source Data file.

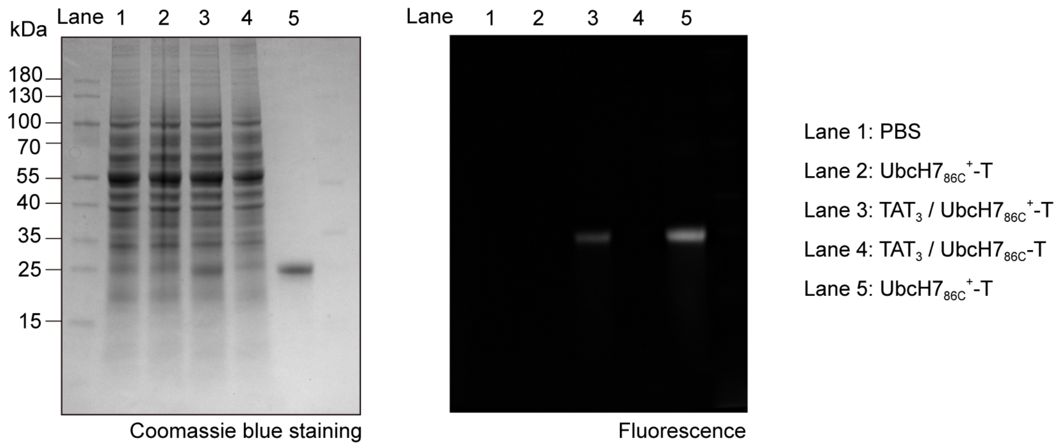


**Supplementary Fig. 7 Coomassie staining and fluorescence images of cell lysates.** HepG2 cells were performed four treatments, respectively. The purified UbcH7$_{86C}$$^{+}$-T was used as controls. The images shown are representative of independent biological replicates (n = 3). Source data are provided as a Source Data file.

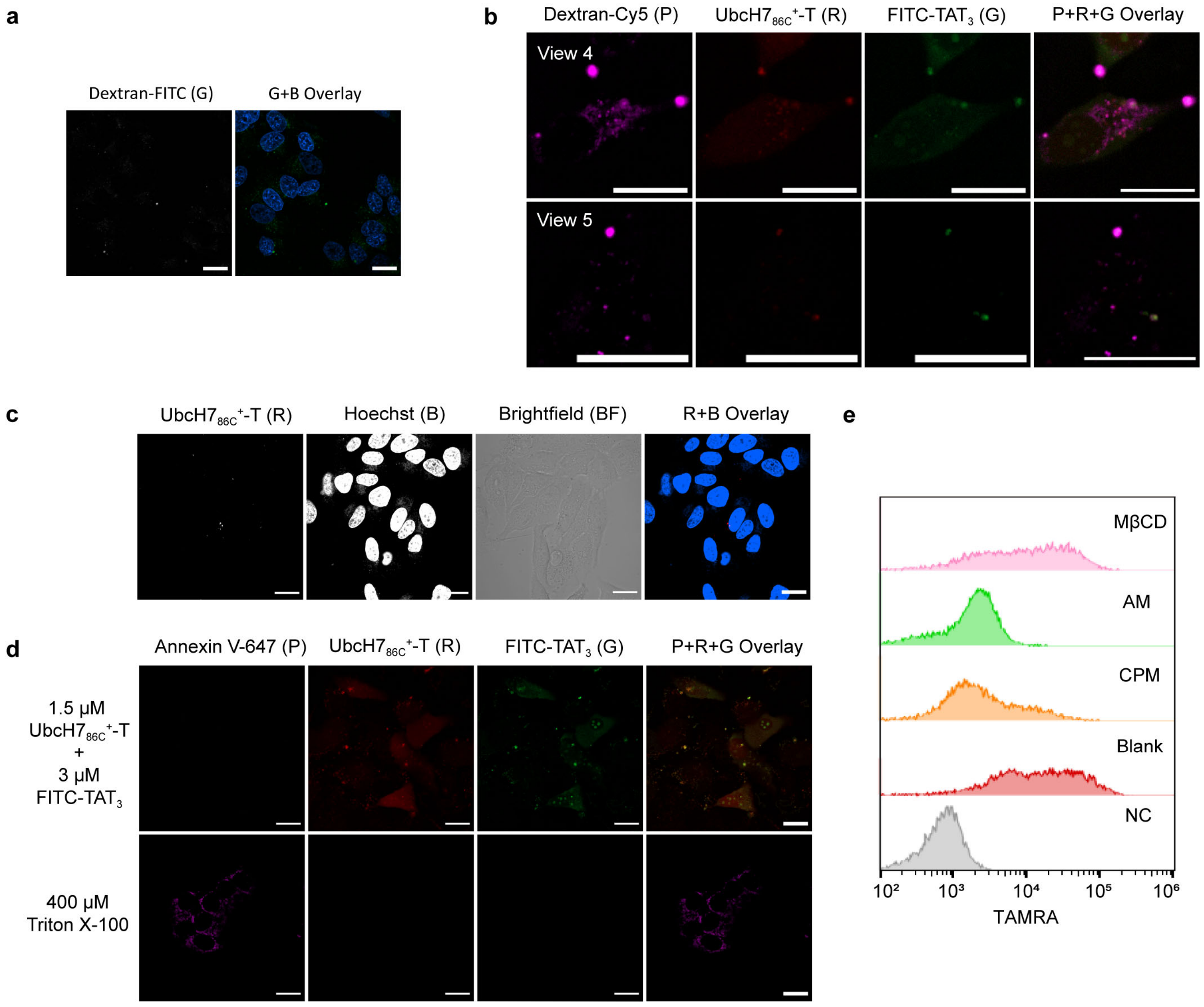


**Supplementary Fig. 8 Additional images of study on internalization mechanism. a,** Confocal microscopy images of HepG2 cells treated with 1 mg/mL 70 kDa dextran-FITC for 30 minutes at 37 °C, followed by washing with PBS containing heparin for imaging. **b,** Additional images of HepG2 cells treated with 1 mg/mL 70 kDa dextran-Cy5, 1.5 μM UbcH7$_{86C}$$^{+}$-T and 3 μM FITC-TAT$_3$ simultaneously at 37 °C for 30 minutes. **c,** Fluorescence images of HepG2 cells treated with 1.5 μM UbcH7$_{86C}$$^{+}$-T and 3 μM TAT$_3$ at 4 °C for 30 minutes. **d,** Fluorescence images of HepG2 cells treated with 1.5 μM UbcH7$_{86C}$$^{+}$-T and 3 μM FITC-TAT$_3$ in the presence of annexin V-647 at 37 °C for 10 minutes. Cells treated with 400 μM Triton-X 100 in the buffer (10 mM HEPES, 140 mM NaCl, 2.5 mM $CaCl_2$, pH 7.4) for 10 minutes were used as positive controls. **e,** Changes in the relative MFI of HepG2 cells pretreated with various endocytosis inhibitors followed by incubation with UbcH7$_{86C}$$^{+}$-T and TAT$_3$ for 30 minutes. "B", "R", "G", "P" and "BF" stand for Hoechst (blue), TAMRA (red), FITC (green), Cy5 (purple) and brightfield signals, respectively. The images shown in Supplementary Fig. 8a-d are representative of independent biological replicates (n = 3). Scale bars, 20 μm.

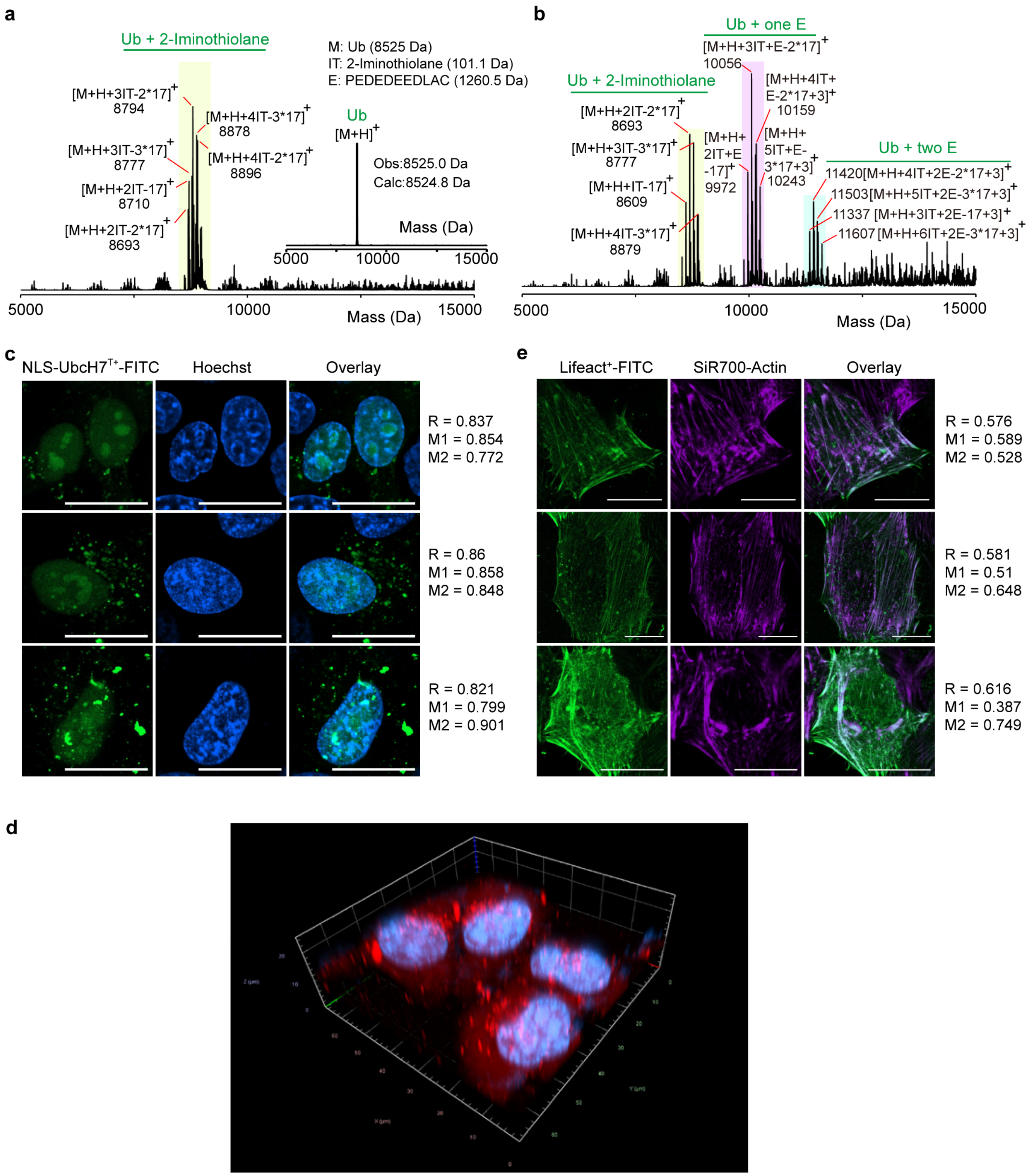


**Supplementary Fig. 9 Additional internalization and co-localization images. a,** Deconvoluted mass characterization of Ub and 2-iminothiolane treated Ub. 17 Da smaller than the desired mass is a by-product of cyclized thiolated Ub that could not be used for following reactions[33]. **b,** Deconvoluted mass characterization of anionic peptide modified Ub. **c,** Whole cell co-localization analysis of NLS-UbcH7$^{+}$-FITC and Hoechst signal. Co-localization images from the left: green channel (NLS-UbcH7$^{+}$-FITC); blue channel (Hoechst). **d,** Z-stack image of HepG2 cells treated with 1.5 μM RFP$^{+}$ and 3 μM $TAT_3$ for 30 minutes at 37 °C. **e,** Whole cell co-localization analysis

of Lifeact$^{+}$-FITC and SiR700-Actin signal. Co-localization images from the left: green channel (Lifeact$^{+}$-FITC); purple channel (SiR700-Actin). The images shown in Supplementary Fig. 9c-e are representative of independent biological replicates (n = 3). Scale bars, 20 μm. Source data are provided as a Source Data file.

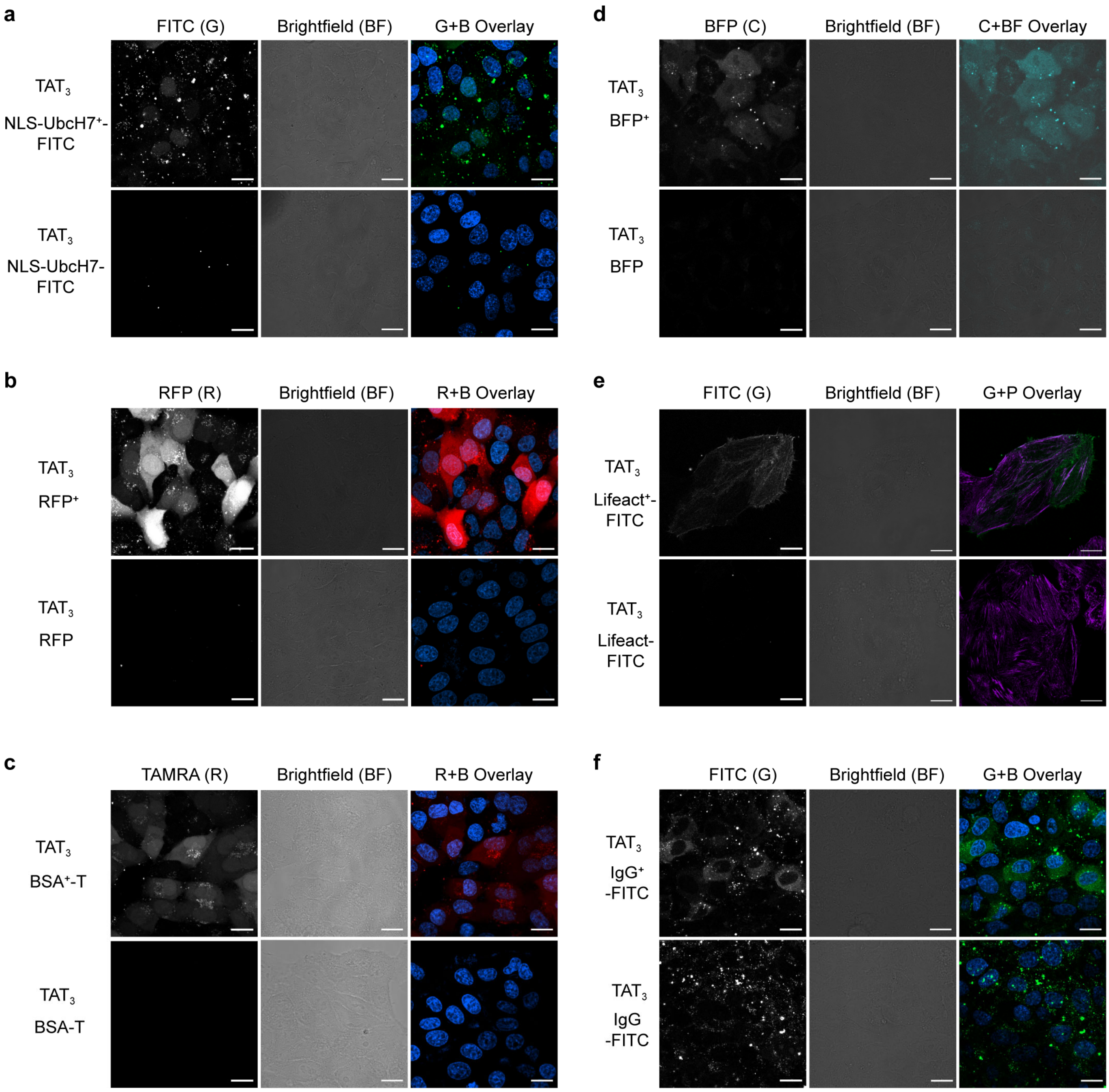


**Supplementary Fig. 10 Cellular uptake of protein cargoes using $TAT_3$. a,** Confocal microscopy images of HepG2 cells treated with 3 μM NLS-UbcH7$^+$-FITC or NLS-UbcH7-FITC in the presence of 3 μM $TAT_3$ for 30 min at 37 °C. **b,** Confocal microscopy images of HepG2 cells treated with 1.5 μM RFP$^+$ or RFP in the presence of 3 μM $TAT_3$ for 30 min at 37 °C. **c,** Confocal microscopy images of HepG2 cells treated with 1.5 μM BSA$^+$-T or BSA-T in the presence of 3 μM $TAT_3$ for 30 min at 37 °C. **d,** Confocal microscopy images of HepG2 cells treated with 1.5 μM BFP$^+$ or BFP in the presence of 3 μM $TAT_3$ for 30 min at 37 °C. **e,** Images of HepG2 cells treated with 3 μM Lifeact$^+$-FITC or Lifeact-FITC in the presence of 3 μM $TAT_3$ for 30 min at 37 °C. **f,** Images of HepG2 cells treated with 1 μM IgG$^+$-FITC or IgG-FITC in the presence of 3 μM $TAT_3$ for 30 min at 37 °C. After incubation, all samples were washed with PBS containing heparin, respectively, for imaging. "B", "R", "G", "C" and "BF" stand for Hoechst (blue), TAMRA (red), FITC (green), BFP (cyan) and brightfield signals, respectively. The images shown in Supplementary Fig. 10a-f are representative of independent biological replicates (n = 3). Scale bars, 20 μm.

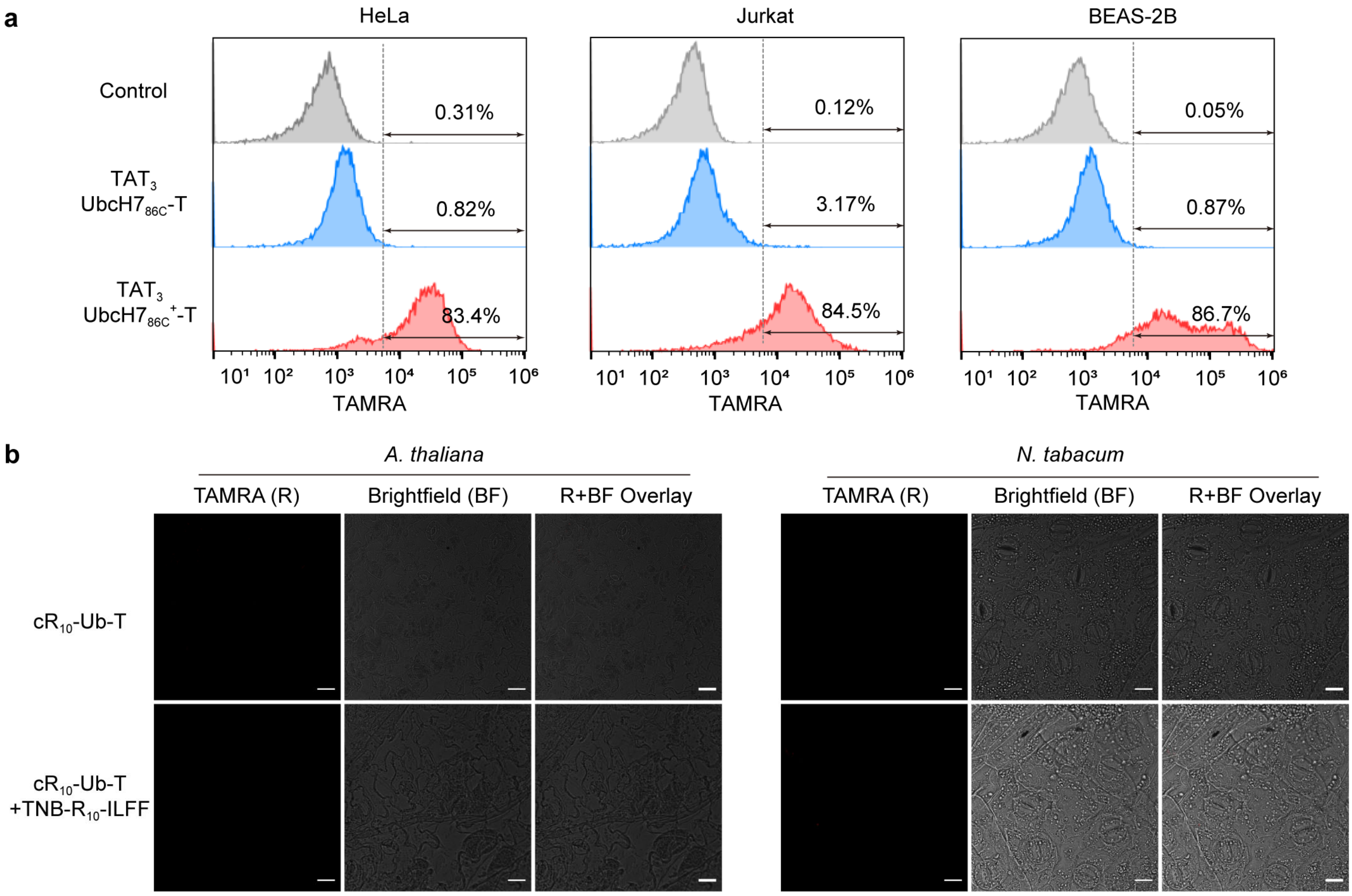


**Supplementary Fig. 11 Cellular uptake of $cR_{10}$-Ub-TAMRA in plants. a,** Flow cytometry analysis of $UbcH7_{86C}{}^{+}$-T uptake in different mammalian cell lines. Mammalian cell lines, including HeLa, BEAS-2B and Jurkat cells were treated with 1.5 μM $UbcH7_{86C}{}^{+}$-T and $UbcH7_{86C}$-T in the presence of $TAT_3$ for 30 minutes at 37 °C, respectively, followed by washing with PBS containing heparin for flow cytometry analysis. **b,** Cellular uptake of $cR_{10}$-Ub-T in plants. Representative images of *A. thaliana* and *N. tabacum* leaves incubated with 3 μM $cR_{10}$-Ub-T alone or in the presence of 5 μM TNB-$R_{10}$-ILFF for 1 hour after treatment with QSY21-NHS. “R” and “BF” stand for TAMRA (red) and brightfield signals, respectively. The images shown are representative of independent biological replicates (n = 3). Scale bars, 20 μm.

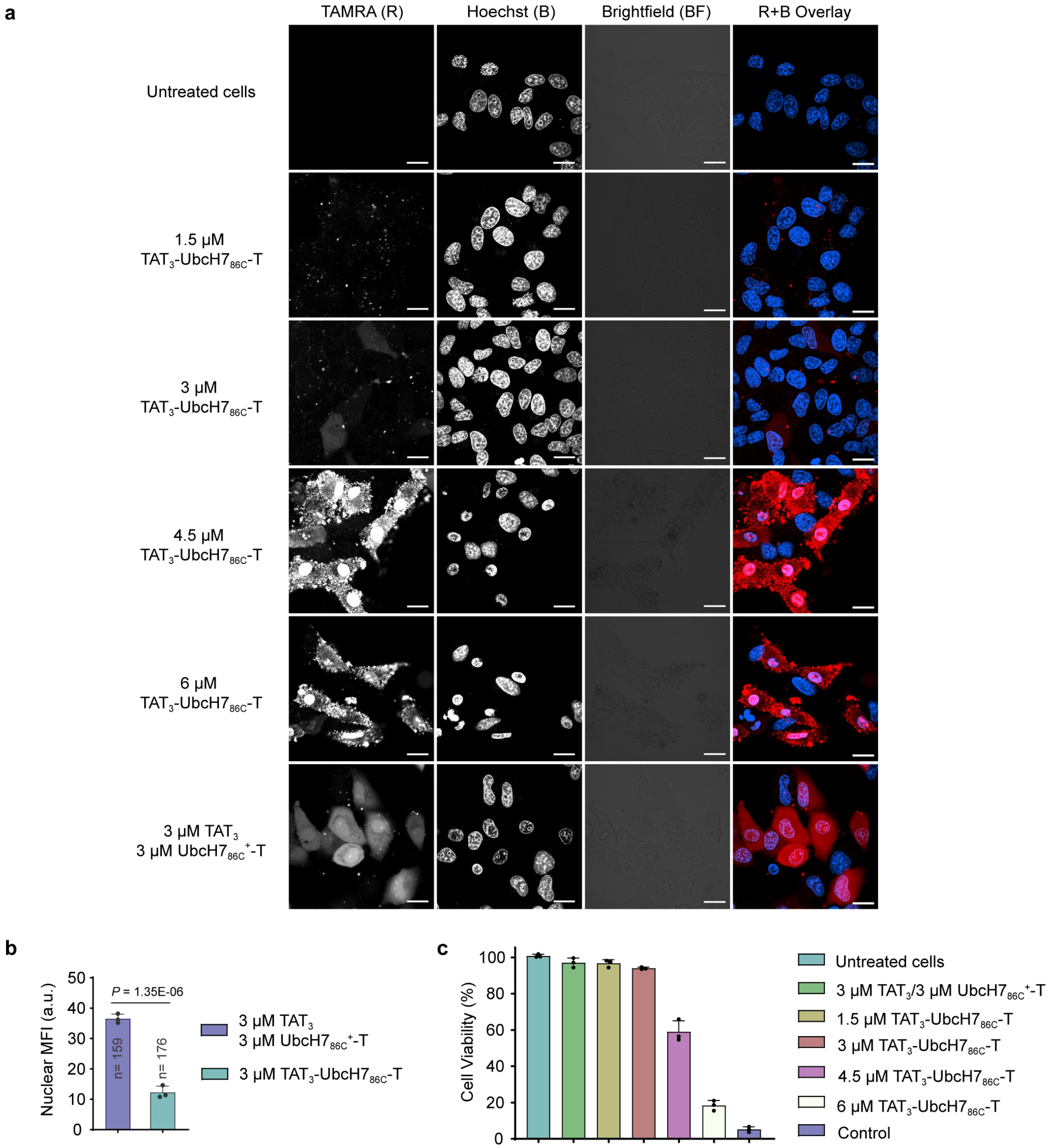


**Supplementary Fig. 12 Cellular uptake of TAT$_3$-UbcH7$_{86C}$-T. a,** Confocal microscopy images of HepG2 cells treated with different concentrations of TAT$_3$-UbcH7$_{86C}$-T or 3 μM UbcH7$_{86C}^{+}$-T in the presence of 3 μM TAT$_3$, for 30 minutes at 37 °C, followed by washing with PBS containing heparin for imaging. **b,** Quantification of the nuclear TAMRA mean fluorescence intensity (MFI) of cells treated with 3 μM TAT$_3$- UbcH7$_{86C}$-T or 3 μM UbcH7$_{86C}^{+}$-T in the presence of 3 μM TAT$_3$. The results are the average of three biological replicates and presented as the mean ± standard deviation. *P* -value was calculated via two-sided t test. **c,** Cell viability detected by a CCK-8 assay after treatment of HepG2 cells with different concentrations of TAT$_3$-UbcH7$_{86C}$-T for 30 minutes in serum-free DMEM. Treatment with 0.64% phenol was used as a control. "B", "R" and "BF" stand for Hoechst (blue), TAMRA

(red) and brightfield signals, respectively. The results are the average of three biological replicates The data are presented as the mean ± standard deviation. Scale bars, 20 µm. Source data are provided as a Source Data file.

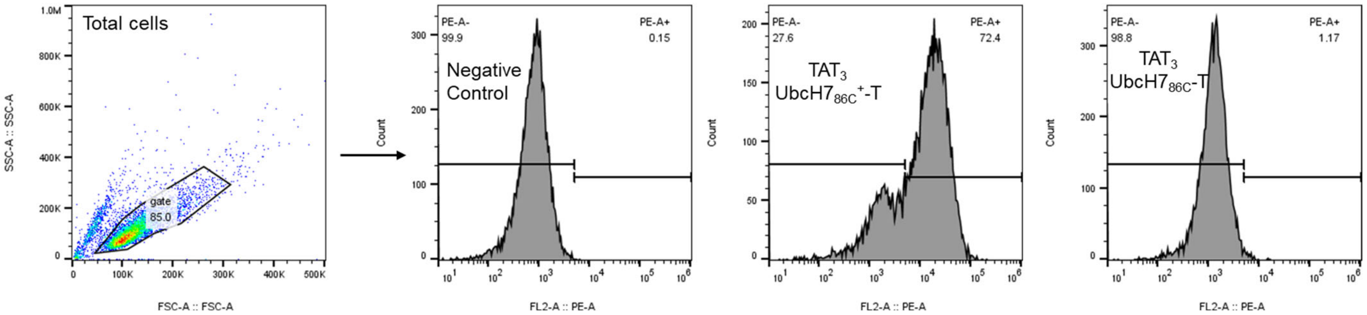


**Supplementary Fig. 13 Gating strategy for flow cytometry data.** Shown is the gating for cells that were treated with medium. Gating panel 3 and 4 corresponds to Fig. 3i.

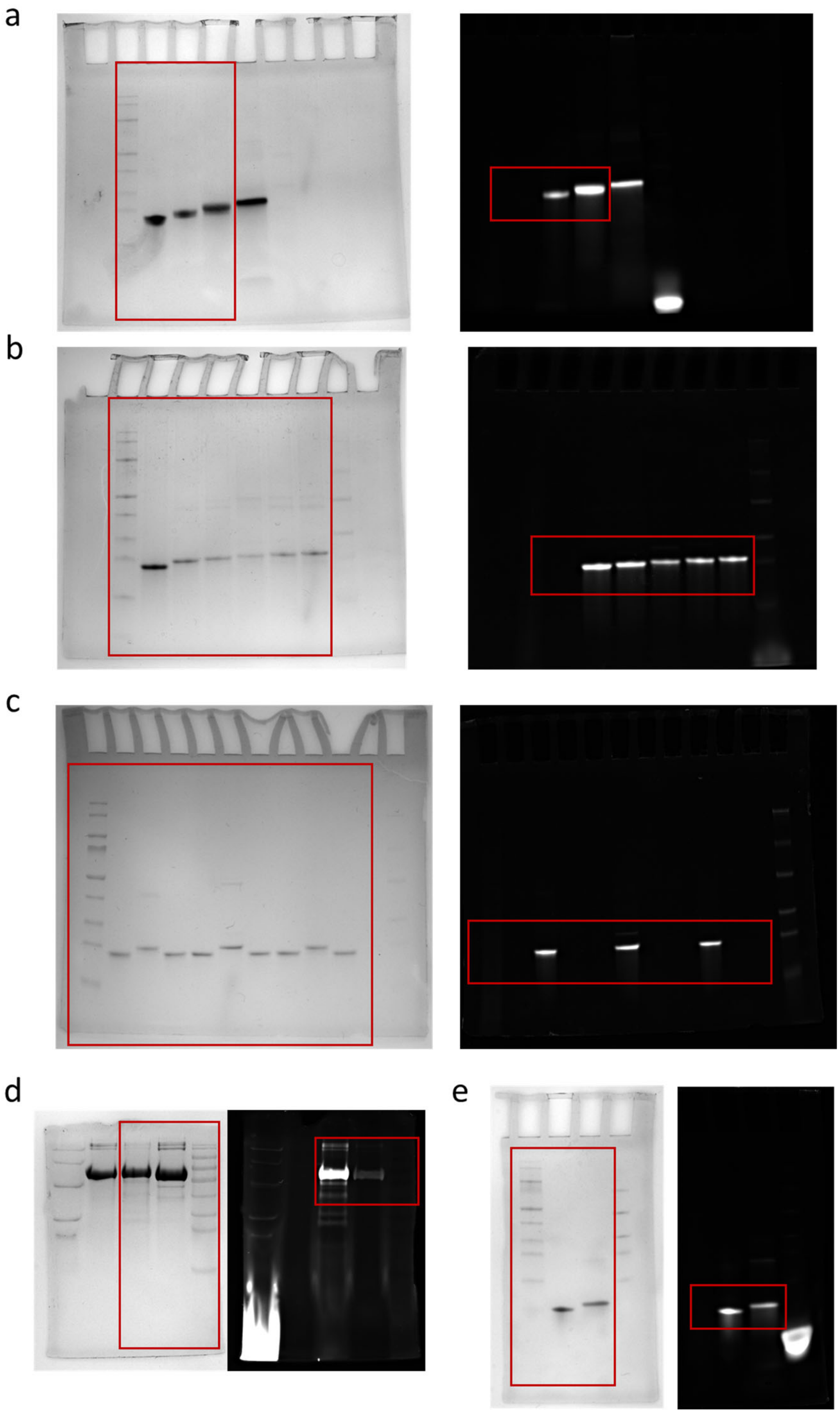
a
b
c
d
e

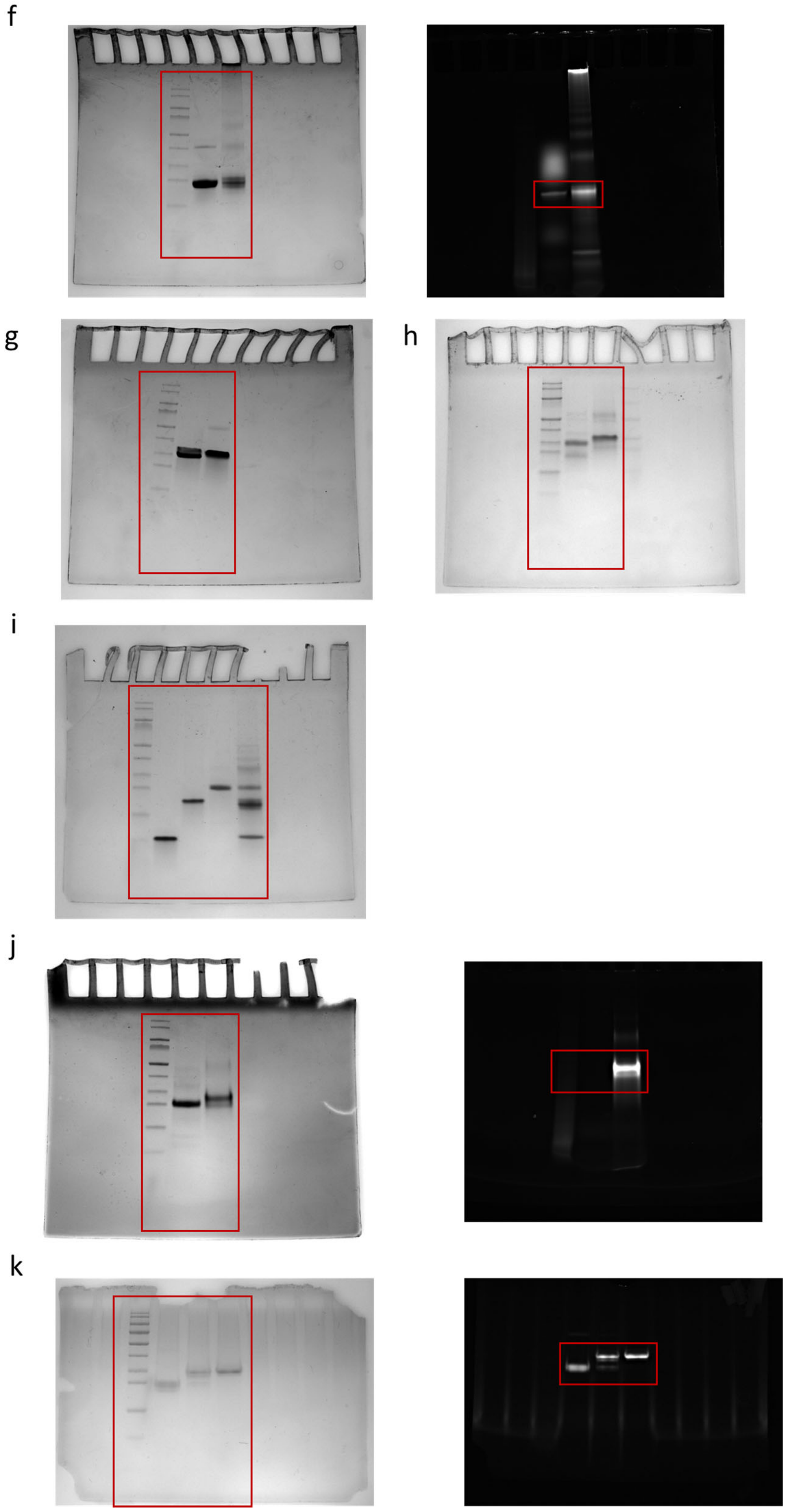


**Supplementary Fig. 14 Unprocessed full scans of SDS-PAGE gels with cropped areas shown in boxes. a-b,** SDS-PAGE gel (Coomassie staining in left panel) and fluorescence image of the same gel (right panel) for Supplementary Fig. 2a and 2b. **c-d,** SDS-PAGE gel (Coomassie staining in left panel) and fluorescence image of the same gel (right panel) for Supplementary Fig. 2e and 2f. **e,** SDS-PAGE gel (Coomassie staining in left panel) and fluorescence image of the same gel (right panel) for Supplementary Fig. 2j. **f,** SDS-PAGE gel (Coomassie staining

in left panel) and fluorescence image of the same gel (right panel) for Supplementary Fig. 2g. **g-h,** SDS-PAGE gel for Supplementary Fig. 2h and 2i. **i,** SDS-PAGE gel for Supplementary Fig. 2k. **j,** SDS-PAGE gel (Coomassie staining in up panel) and fluorescence image of the same gel (down panel) for Supplementary Fig. 2l. **k,** SDS-PAGE gel (Coomassie staining in up panel) and fluorescence image of the same gel (down panel) for Supplementary Fig. 2m.

**Supplementary Table 1. Identified RING E3s in our work.** RING E3s found in EGF-stimulated living HeLa cells and their corresponding partner E2 are listed here. "/" indicates there is no literature about their corresponding partner E2. For each protein, the fold change and adjusted *P*-value from analysis are provided. Only proteins met the criteria of *P*-value < 0.05 (Benjamini-Hochberg correction for multiple comparisons) and fold change > 2 were considered significantly enriched. Statistical significance was determined by a two-sided t-test.

| Gene name | Fold charge | *P*-value | Partner E2 | Reference |
|---|---|---|---|---|
| ZNF598 | 17.03117 | 2.56000E-05 | UBE2D3 | *Nat. Commun.* 8, 16056 (2017). |
| BRAP | 28.20887 | 3.58400E-03 | UBE2D3 | *Biochem. J.* 474, 3207-3226 (2017). |
| RN168 | 45.40402 | 1.12000E-04 | UBE2D3 | *Cell* 150, 1182-1195 (2012). |
| RBBP6 | 10.43105 | 4.82000E-05 | UBE2D3 | *Cell Rep.* 7, 575-587 (2014). |
| MID1 | 0.426002 | 3.90000E-04 | UBE2D3 | *J. Biol. Chem.* 289, 31805-31817 (2014). |
| UHRF1 | 6.699226 | 8.72000E-06 | UBE2D3 | *Mol. Cell* 72, 753-765.e6 (2018). |
| RNF126 | 37.11017 | 7.48000E-06 | UBE2D3 | *Cell Rep.* 18, 2007-2017 (2017). |
| RNF10 | 3.665142 | 6.47800E-03 | UBE2D3 | *Cell Rep.* 36, 109642 (2021). |
| RING1 | 17.14389 | 6.70600E-02 | UBE2D3 | *Proc. Natl. Acad. Sci. U.S.A.* 115, 1558-1563 (2018). |
| CBL | 1.541995 | 5.56233E-01 | UBE2D3 | *Mol. Biol. Evol.* 37, 1986-2001 (2020). |
| TRIM25 | 5.376635 | 2.21860E-02 | UBE2D3 | *Cell Rep.* 16, 1315-1325 (2016). |
| TRIM32 | 20.39823 | 1.63000E-05 | UBE2D3 | *J. Mol. Biol.* 354, 413-424 (2005). |
| UHRF2 | 18.47128 | 1.15200E-03 | UBE2D3 | *Mol. Cell* 72, 753-765.e6 (2018). |
| TRIM33 | 1.521253 | 4.88251E-01 | UBE2D3 | *Nat. Commun.* 6, 6156 (2015). |
| RNF167 | 1.399463 | 5.07637E-01 | UBE2D3 | *FEBS J.* 288, 4849-4868 (2021). |
| ZNRF2 | 0.888425 | 7.16468E-01 | UBE2D3 | *J. Neurosci.* 23, 9385-9394 (2003). |
| RNF138 | 0.542205 | 5.01607E-01 | UBE2D3 | *J. Cancer Res. Ther.* 19, 1636-1645 (2023). |
| RNF181 | 9.608539 | 7.38000E-04 | UBE2D3 | *Mol. Cell Biol.* 36, 794-808 (2016). |
| MID2 | 0.750968 | 5.76785E-01 | UBE2D3 | *Biochem. J.* 434, 309-319 (2011). |
| TRIM11 | 1.294959 | 3.50528E-01 | UBE2D3 | *Biochem. J.* 434, 309-319 (2011). |
| RNF114 | 86.21266 | 3.46000E-05 | UBE2D1 | *Sci. Adv.* 9, eadg7752 (2023). |
| RNF169 | 2.992554 | 2.18300E-03 | UBE2D1 | *J. Cell Biol.* 197, 189-199 (2012). |
| RNF25 | 54.44943 | 1.21000E-04 | UBE2D2 | *J. Biol. Chem.* 290, 30225-30239 (2015). |
| XIAP | 26.04659 | 2.44000E-07 | UBE2D1、UBE2D2 | *Proc. Natl. Acad. Sci. U.S.A.* 98, 8662-8667 (2001). |
| CBLB | 0.683809 | 7.67900E-03 | UBE2D2 | *Nat. Med.* 22, 915-923 (2016). |
| ZFP91 | 0.025734 | 1.89000E-03 | UBE2D1 | *Nat. Commun.* 8, 15398 (2017). |
| RNF220 | 12.04226 | 2.12000E-04 | UBE2E1 | *Biochem. Bioph. Res. Co.* 393, 708-713 (2010). |
| RNF115 | 12.00649 | 1.14660E-02 | UBE2D1 | *Front. Immunol.* 13, 936579 (2022). |
| RFWD3 | 4.971478 | 7.97200E-03 | UBE2D1 | *Proc. Natl. Acad. Sci. U.S.A.* 107, 4579-4584 (2010). |
| MIB1 | 0.330842 | 1.43380E-02 | UBE2D2 | Preprint at https://doi.org/10.1101/2024.03.01.582834 (2024). |
| PJA2 | 0.733242 | 4.10352E-01 | UBE2D2 | *Genomics* 79, 869-874 (2002). |
| TRIM7 | 0.752653 | 5.77606E-01 | UBE2D1 | *Nature* 585, 414-419 (2020). |
| AMFR | 0.508572 | 2.23460E-01 | UBE2D1 | *Immunity* 41, 919-933 (2014). |
| TRIM56 | 0.656767 | 5.32532E-01 | UBE2D1 | *Comput. Struct. Biotec.* 21, 2801-2808 (2023). |
| MARCHF5 | 2.538118 | 1.64503E-01 | UBE2D2 | *Mol. Cell* 77, 1107-1123.e10 (2020). |
| DZIP3 | 0.633622 | 1.31940E-01 | UBE2E1 | *Nat. Commun.* 4, 2939 (2013). |
| MKRN2 | 1.750929 | 1.03260E-02 | UBE2L3 | *Biochem. Bioph. Res. Co.* 529, 43-50 (2020). |

| Gene name | Fold charge | *P*-value | Partner E2 | Reference |
|---|---|---|---|---|
| RAD18 | 8.058684 | 1.98237E-01 | UBE2A | *Nucleic Acids Res.* 35, 5819-5830 (2007). |
| TTC3 | 6.403628 | 3.89000E-05 | UBE2E3 | *Cell Death Dis.* 10, 92 (2019). |
| RNF20 | 0.713946 | 3.82408E-01 | UBE2B | *Mol. Cell* 41, 515-528 (2011). |
| RNF123 | 2.684284 | 9.56000E-05 | / | / |
| TRIM47 | 2.305065 | 1.55150E-02 | / | / |

**Supplementary Table 2. The $log_2$LFQ fold difference of significantly enriched RING E3s**. Experimental cells (A) were then incubated with probe$^+$ and $TAT_3$, while control cells (B) were incubated with probe$^+$ alone. Proteins with a *P*-value < 0.05 (Benjamini-Hochberg correction for multiple comparisons) and fold change > 2 were considered significantly enriched corresponding to Fig. 7d. Statistical significance was determined by a two-sided t-test. LFQ: Label-free quantification.

| | | A | | | B | |
|---|---|---|---|---|---|---|
| Gene name | $log_2$LFQ A1 | $log_2$LFQ A2 | $log_2$LFQ A3 | $log_2$LFQ B1 | $log_2$LFQ B2 | $log_2$LFQ B3 |
| RNF126 | 25.7329 | 25.59017 | 25.63296 | 21.41135 | 18.39569 | 20.11061 |
| UHRF1 | 24.82087 | 24.8477 | 24.89748 | 22.52833 | 21.90202 | 21.79063 |
| TRIM32 | 23.18728 | 23.13594 | 23.23029 | 17.13784 | 20.08471 | 17.15893 |
| ZNF598 | 28.1562 | 28.12881 | 27.96106 | 24.39423 | 24.03642 | 23.37564 |
| RBBP6 | 25.16414 | 25.20692 | 25.11805 | 22.20673 | 22.4446 | 17.98612 |
| RN168 | 25.79829 | 26.1196 | 26.0316 | 20.46436 | 20.56171 | 20.42449 |
| RNF181 | 21.7426 | 21.40443 | 21.83978 | 18.10766 | 17.30094 | 19.19647 |
| UHRF2 | 21.80944 | 21.54792 | 22.11145 | 18.1136 | 16.63 | 17.78102 |
| BRAP | 27.66152 | 27.2395 | 26.87913 | 21.31295 | 23.10724 | 22.48683 |
| RNF10 | 24.73716 | 25.32735 | 25.28042 | 23.84375 | 22.73712 | 22.9654 |
| TRIM25 | 21.22899 | 20.17039 | 21.3706 | 18.38864 | 18.58757 | 18.75952 |
| XIAP | 27.65931 | 27.60244 | 27.60916 | 23.11111 | 23.11685 | 22.4338 |
| RNF114 | 27.07668 | 26.97827 | 27.21581 | 19.91206 | 21.83021 | 18.03281 |
| RNF123 | 25.92087 | 25.95259 | 25.90504 | 24.58496 | 24.69433 | 24.17595 |
| RNF25 | 25.29395 | 25.57845 | 25.30917 | 19.65235 | 17.89907 | 20.38658 |
| RNF169 | 23.63741 | 23.78252 | 23.80219 | 21.10523 | 22.58585 | 22.396 |
| RFWD3 | 20.79137 | 21.08915 | 20.2691 | 18.84601 | 18.4331 | 17.88938 |
| RNF115 | 22.44336 | 22.93167 | 21.87576 | 19.59761 | 18.73193 | 17.83082 |
| TTC3 | 21.22915 | 21.22818 | 21.20144 | 19.00465 | 17.46772 | 18.73665 |
| RNF220 | 22.08997 | 22.31639 | 22.40546 | 16.66836 | 18.93857 | 19.33026 |
| TRIM47 | 22.81219 | 22.43616 | 22.23991 | 21.2396 | 21.705 | 20.8659 |

**Supplementary Table 3. Protein sequences.** The Amino acid sequence of proteins used in the work are list as follows.

| Protein name | Amino acid sequence |
|---|---|
| BFP (Blue fluorescent protein) | MHHHHHHMVSKGEELIKENMHMKLYMEGTVDNHHFKCTSEGEGKPYEGTQTMRIKV<br>VEGGPLPFAFDILATSFLYGSKTFINHTQGIPDFFKQSFPEGFTWERVTTYEDGGVLTAT<br>QDTSLQDGCLIYNVKIRGVNFTSNGPVMQKKTLGWEAFTETLYPADGGLEGRNDMAL<br>KLVGGSHLIANAKTTYRSKKPAKNLKMPGVYYVDYRLERIKEANNETYVEQHEVAVA<br>RYCDLPS KLGHKLN* |
| RFP (Red fluorescent protein) | MSELIKENMHMKLYMEGTVNNHHFKCTSEGEGKPYEGTQTMRIKVVEGGPLPFAFDIL<br>ATSFMYGSRTFINHTQGIPDFFKQSFPEGFTWERVTTYEDGGVLTATQDTSLQDGCLIY<br>NVKIRGVNFPSNGPVMQKKTLGWEANTEMLYPADGGLEGRSDMALKLVGGGHLICNF<br>KTTYRSKKPAKNLKMPGVYYVDHRLERIKEADKETYVEQHEVAVARYCDLPSKLGHK<br>LEHHHHHH |
| UbcH7 | MHHHHHHMAASRRLMKELEEIRKCGMKNFRNIQVDEANLLTWQGLIVPDNPPYDKG<br>AFRIEINFPAEYPFKPPKITFKTKIYHPNIDEKGQVCLPVISAENWKPATKTDQVIQSLIAL<br>VNDPQPEHPLRADLAEEYSKDRKKFCKNAEEFTKKYGEKRPVDG* |
| UbcH7$_{C17}$ | MHHHHHHMAASRRLMKELEEIRKCGMKNFRNIQVDEANLLTWQGLIVPDNPPYDKG<br>AFRIEINFPAEYPFKPPKITFKTKIYHPNIDEKGQVALPVISAENWKPATKTDQVIQSLIAL<br>VNDPQPEHPLRADLAEEYSKDRKKFAKNAEEFTKKYGEKRPVDG* |
| UbcH7$_{C86}$ | MHHHHHHMAASRRLMKELEEIRKAGMKNFRNIQVDEANLLTWQGLIVPDNPPYDKG<br>AFRIEINFPAEYPFKPPKITFKTKIYHPNIDEKGQVCLPVISAENWKPATKTDQVIQSLIAL<br>VNDPQPEHPLRADLAEEYSKDRKKFAKNAEEFTKKYGEKRPVDG* |
| UbcH7$_{C137}$ | MHHHHHHMAASRRLMKELEEIRKAGMKNFRNIQVDEANLLTWQGLIVPDNPPYDKG<br>AFRIEINFPAEYPFKPPKITFKTKIYHPNIDEKGQVALPVISAENWKPATKTDQVIQSLIAL<br>VNDPQPEHPLRADLAEEYSKDRKKFCKNAEEFTKKYGEKRPVDG* |
| NLS-UbcH7 | MIPKKKRKVAASRRLMKELEEIRKCGMKNFRNIQVDEANLLTWQGLIVPDNPPYDKG<br>AFRIEINFPAEYPFKPPKITFKTKIYHPNIDEKGQVCLPVISAENWKPATKTDQVIQSLIAL<br>VNDPQPEHPLRADLAEEYSKDRKKFCKNAEEFTKKYGEKRPVDGHHHHHH* |
| UBE2D3 C85KS22R | MHHHHHHMALKRINKELSDLARDPPAQCRAGPVGDDMFHWQATIMGPNDSPYQGGV<br>FFLTIHFPTDYPFKPPKVAFTTRIYHPNINSNGSIKLDILRSQWSPALTISKVLLSICSLLCD<br>PNPDDPLVPEIARIYKTDRDKYNRISREWTQKYAM* |
